# Does Caplacizumab for the management of thrombotic thrombocytopenic purpura increase the risk of relapse, exacerbation, and bleeding? An updated systematic review and meta‐analysis based on revised criteria by the International Working Group for thrombotic thrombocytopenic purpura

**DOI:** 10.1002/jha2.833

**Published:** 2023-12-18

**Authors:** Niraj Neupane, Sangharsha Thapa, Amir Mahmoud, Abhinav Bhattarai, Anil KC, Shreeja Shikhrakar, Sayuri Gurusinghe, Peter Kuiodes

**Affiliations:** ^1^ Rochester General Hospital Department of Internal Medicine Rochester New York USA; ^2^ Kathmandu University School of Medical Sciences Dhulikhel Nepal; ^3^ Institute of Medicine, Maharjgunj Medical Campus Kathmandu Nepal; ^4^ Patan Academy of Health Sciences Patan Nepal; ^5^ University of Buffalo Department of Internal Medicine Buffalo New York USA; ^6^ Lipson Cancer Center, Rochester General Hospital Department of Hematology New York USA

## Abstract

Thrombotic thrombocytopenic purpura (TTP) is a rare and life‐threatening condition marked by abnormal blood clotting and organ damage. Caplacizumab is a potential treatment for the TTP management. This systematic review and meta‐analysis aimed to assess Caplacizumab's effectiveness and safety in the TTP management. A comprehensive database search identified nine studies, including randomized controlled trials and observational studies. Primary outcomes included TTP exacerbation, relapse, and major bleeding. Major bleeding risk was evaluated using updated definitions recommended by the International TTP Working Group in 2021. Revised criteria proposed by the IWG for TTP recurrence were employed for a comprehensive assessment of Caplacizumab's impact on relapse and exacerbation. Analysis revealed Caplacizumab significantly reduced all‐cause mortality in TTP patients. Some studies raised concerns about bleeding risk, but overall, it did not significantly differ from standard treatment. Likewise, there was no significant difference in TTP relapse rates between Caplacizumab and standard care. This study supports Caplacizumab as a potential adjunct therapy for TTP. However, careful consideration of its advantages and risks is crucial in clinical practice. Further research is needed to address concerns related to adverse effects like bleeding risk and relapse rates associated with Caplacizumab in the TTP management. The findings emphasize the importance of weighing potential benefits and risks when considering Caplacizumab as an adjunct therapy for TTP.

## 1 INTRODUCTION

Thrombotic thrombocytopenic purpura (TTP) is a rare and life‐threatening autoimmune thrombotic microangiopathy that causes hemolytic anemia, thrombocytopenia, and organ damage [1]. TTP is caused by the deficiency or dysfunction of ADAMTS13, an enzyme that cleaves ulVWF, which, if in excess, leads to spontaneous platelet aggregation. This results in the accumulation of uncleaved ultra‐large von Willebrand factor (vWF) multimers, which are highly thrombogenic and promote the formation of microthrombi [2]. The management of TTP requires prompt diagnosis and treatment. Following the introduction of plasma exchange (PEx) therapy, the mortality rate of TTP dropped significantly from more than 90% to less than 20% [3]. PEx, corticosteroids, and early use of rituximab have become the standard treatment for TTP. In recent years, the anti‐von Willebrand factor monoclonal antibody, Caplacizumab, has been deployed to manage TTP with the promise of further improving recovery and survival. Clinical trials have shown that this treatment statistically reduces the time to platelet count normalization and the number of TTP‐related complications [1, 4].

The mechanism of Caplacizumab is inhibition of the interaction at the A1 domain of von Willebrand factor binding to platelet glycoprotein receptor 1b. This limits platelet adhesion/aggregation and subsequent microvascular thrombus formation [5]. In the Phase II TITAN trial, Caplacizumab significantly reduced the time to platelet count normalization, duration of PEx, and several TTP‐related complications, such as neurological events [1]. The Phase III HERCULES trial also supported the efficacy of Caplacizumab in reducing the time to platelet count normalization and TTP‐related complications [4]. Overall, these clinical trials provide evidence of Caplacizumab as a promising, new adjunct therapy when managing this rare and life‐threatening autoimmune disorder.

However, Caplacizumab has also been associated with several adverse effects in treating TTP. Aside from the more common side effects like nausea, vomiting, headache, and non‐threatening mucocutaneous bleeding, there is also a risk of serious side effects such as life‐threatening hemorrhages due to reduced vWF levels from Caplacizumab [6]. Coupled with the high cost, some authors have expressed concern over the net clinical benefit of Caplacizumab in TTP [7]. Interestingly, most bleeding events recorded in the TITAN and HERCULES study were mild to moderate and did not require therapeutic interventions [1]. Despite this, reports of catheter site hemorrhage, intracranial hemorrhage, and gastrointestinal bleeding in real‐world studies have raised concerns indicating the need for further investigation of the safety of Caplacizumab [8, 9]. Moreover, clinical trials have shed light on the potential of Caplacizumab to augment relapse.

A recent systematic review and meta‐analysis of Randomised Clinical trials (RCTs) and observational studies has suggested a significant rise in relapse rates in Caplacizumab treated patients compared to the standard of care (SOC) alone [10]. However, most studies have used the older terminology for reporting TTP recurrences, where durable remission was considered to occur after 30 days of remission from PEx [11]. This does not consider the temporizing effect of Caplacizumab (usually taken for 30 days) on platelet counts and thus misrepresents early recurrences after stoppage of Caplacizumab as relapses rather than exacerbations. This is better accounted for by the new criteria proposed by the International TTP Working Group in 2021, where recurrences within 30 days of stoppage of PEx or Caplacizumab are considered exacerbations, which we have adhered to and implemented in our study.

To accomplish this, we attempted to extract data from RCTs and observational studies that reported the exact timing of TTP recurrence in relation to the treatment protocols and redefined the exacerbation/relapse endpoints to reflect the new definition. We conducted a systematic review and meta‐analysis to evaluate the effect of Caplacizumab more accurately on TTP recurrence consistent with the post‐Caplacizumab era. We also focused on the risk of major bleeding associated with the addition of Caplacizumab compared to SOC alone as our safety outcome of interest.

## 2 Methods

This systematic review adheres to standard guidelines and follows the Preferred Reporting Items for Systematic Reviews and Meta‐analyses (PRISMA) statement (12).

### 2.1 Eligibility criteria

The inclusion criteria were as follows:

1. Non‐pregnant adults (≥18 years) with an acute episode of TTP who meet the TTP diagnostic criteria.
2. We included RCTs and nonrandomized observational studies that compared the SOC with and without the addition of Caplacizumab.
3. We included studies that enrolled children and adults if > 90% of the studied population were adults.

The exclusion criteria were as follows:

1. We excluded case reports, case series, and single‐arm studies evaluating Caplacizumab without a comparator group or studies that enrolled patients with known congenital TTP.
2. In vitro and animal experiments
3. Small sample size studies (less than 10 participants)
4. Articles published in languages other than English.

### 2.2 Outcomes

Caplacizumab was the intervention modality within the experimental group, with the SOC alone as our control group. Our primary efficacy outcomes were TTP exacerbation, defined as clinical recurrence within 30 days of stoppage of either PEx or Caplacizumab and TTP relapse, defined as clinical recurrence after 30 days of stoppage of PEx (control group) or Caplacizumab (intervention group); major bleeding was our main safety measure. For the primary efficacy outcome, since most studies reported exacerbation/relapse using the older definition, we extracted the data only from RCTs and observational studies that reported the exact timing of TTP recurrence in relation to the treatment protocols and redefined the exacerbation/relapse endpoints to reflect the above definition. We also assessed the time to platelet count recovery, mortality, refractory TTP, duration of PEx, hospital length of stay (LOS), and treatment‐emergent thrombosis as secondary outcomes. The definition of the outcome has been explained in Table 2.

### 2.3 Information sources

We conducted a systematic literature search on PubMed, Embase, Cochrane, and CHINAL databases from their establishment until October 2023.

### 2.4 Search strategy

Our search used keywords such as “thrombocytopenic purpura,” “thrombotic,” “Moschkowitz disease,” “congenital thrombotic thrombocytopenic purpura,” “Schulman‐Upshaw syndrome,” “Upshaw factor,” “microangiopathic hemolytic anemia,” “congenital,” “thrombotic microangiopathy familial,” “familial thrombotic thrombocytopenic purpura,” “Caplacizumab,” “Cablivi,” “Caplacizumab—yhdp,” and “ALX‐0081”. We also used terms related to drug‐related side effects, adverse reactions, toxicity, and bleeding.

### 2.5 Data collection process

After removing duplicates, each citation was imported into Covidence systematic review software, Veritas Health Innovation, Melbourne, Australia. (Available at www.covidence.org). Two authors independently assessed each reference and abstract according to our selection criteria, and any references of included articles were manually searched.

### 2.6 Data items

Data extraction was performed by two authors independently on Microsoft Excel version 2016. We collected information on the trial setup, eligibility requirements, enrollment time, baseline study participants’ characteristics, available therapies, and important outcomes regarding efficacy and safety. The third author examined the extracted data, and any discrepancies were resolved by discussion with other authors. We extracted event rates for binary outcomes and means or medians for continuous outcomes. If multiple rates were provided, we used the event rates from the most extended follow‐up period.

### 2.7 Risk of bias assessment

Since the included studies were RCTs as well as observational studies, two different models were used to assess the risk of bias. First, the bias across the RCTs was assessed using Cochrane's risk of bias assessment tool for RCTs consisting of seven domains: [1] random sequence generation, [2] allocation concealment, [3] blinding of participants and personnel, [4] blinding of outcome assessment, [5] incomplete outcome data, [6] selective reporting, and [7] other bias [13]. Similarly, bias across the observational studies was reported concerning the Newcastle–Ottawa scale, which consists of three domains: [1] selection of study groups, [2] comparability, and [3] ascertainment of the outcome of interest. Studies are analyzed for each domain, and starts are assigned as points (0–4, 0–2, and 0–3, respectively, for the domains); thus, the final score is rated out of 9 [14].

### 2.8 Effect measures and synthesis methods

We performed this meta‐analysis using an intention‐to‐treat analysis for efficacy outcomes. Data were entered and analyzed using RevMan (version 5.3, Cochrane Collaboration, Oxford, UK). The weighted mean difference (WMD) was used for continuous outcomes (platelet count normalization time, days of plasma exchange, and length of hospital stay) to evaluate the differences between the Caplacizumab treatment and control groups in the included studies. The precision of the effect sizes was reported as 95% confidence intervals (CIs). A pooled estimate of the WMD was computed using the random‐effects model. Relative risk (RR) values and corresponding 95% CIs were used for dichotomous variables such as relapse, mortality, and major thrombotic events. Statistical heterogeneity between the studies was assessed using the *Q* and *I* ^2^ statistics. Pooled effect sizes were generated using a random‐effects model.

## 3 RESULTS

### 3.1 Study selection and characteristics

**FIGURE 1:**
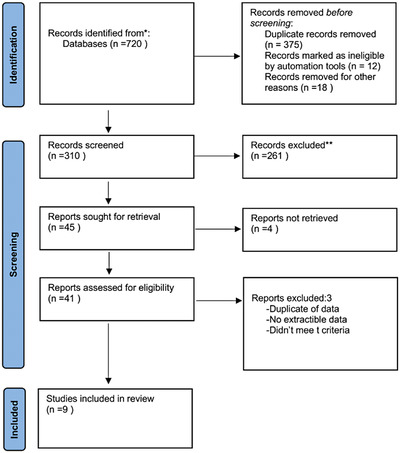
PRISMA guidelines for study selection. PRISMA, Preferred Reporting Items for Systematic Reviews and Meta‐analyses.

The study selection process results are shown in the PRISMA flowchart in Figure 1. Briefly, 720 results were retrieved from the preliminary database search. Then, after removing duplicates, followed by the title and abstract screening, the full text of 41 studies was assessed to fulfill the eligibility criteria. Finally, two RCTs [1, 2, 3, 4] and seven observational studies review [15, 16, 17, 18, 19] nine studies were selected for review.

### 3.2 Risk of bias assessment

**FIGURE 2:**
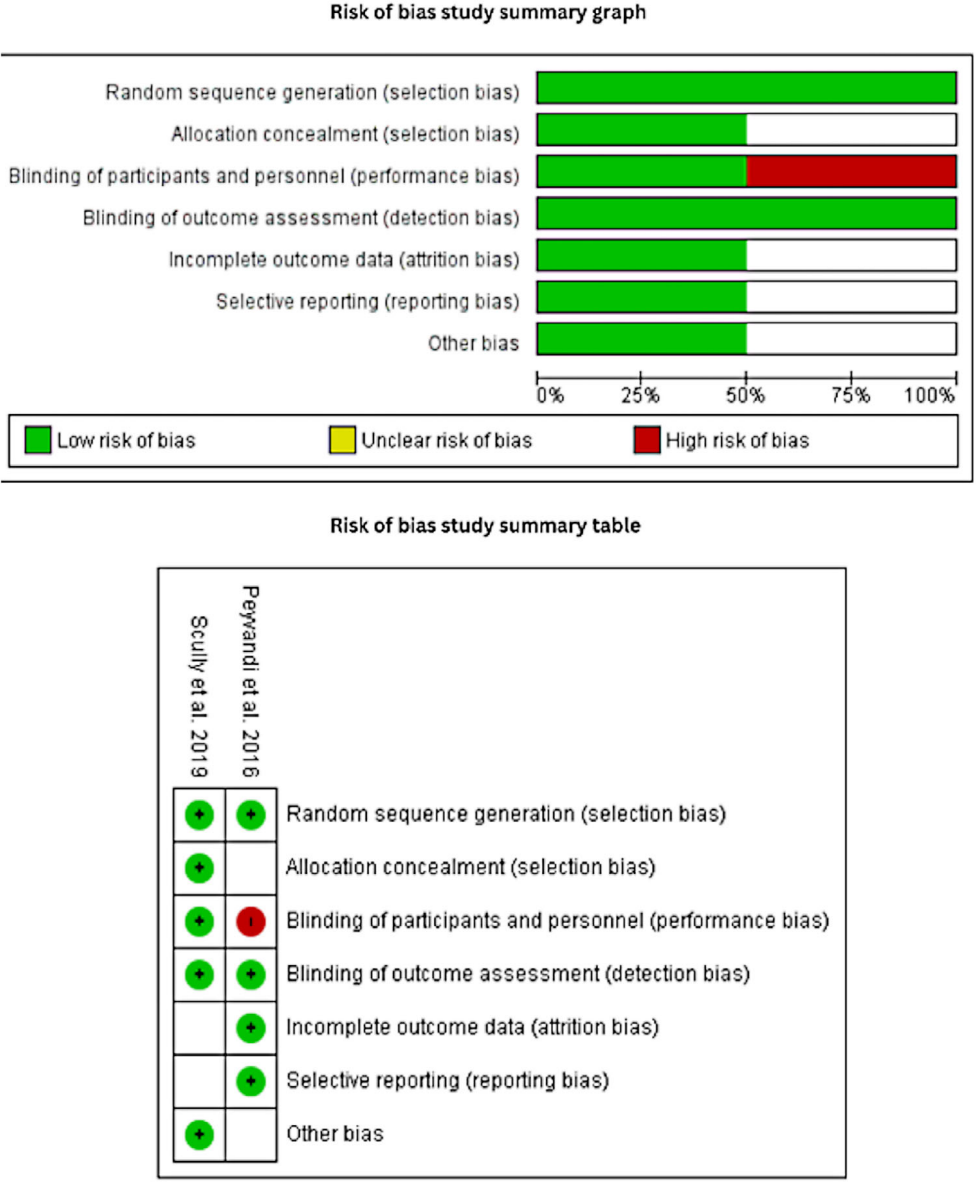
Risk of bias assessment.

There were two RCTs included in this study. The risk of bias assessment results of RCTs is summarized in Figure 2. In both RCTs, the random sequence generation domain had a low bias risk. Since Payvandi et al.’s study was single‐blinded, we marked its blinding of participants and personnel domain as high risk. The bias was marked unclear when the information was inadequate to decide a higher or lower risk. Table 1 shows the results of the risk of bias across the observational studies. Overall, all studies had high quality and a low risk of bias. The total score ranged from 7 to 9.

**TABLE 1:** The results of the risk of bias across the observational studies.

	Selection				Comparability	Outcome			
Study	Representativeness of the exposed cohort.	Selection of the non‐exposed cohort.	Ascertainment of exposure.	Showing that the outcome of interest was not present at the time of the study.	Comparability of the cohorts based on the designs or analysis.	Assessment of outcome.	Follow‐up long enough for the outcomes to occur.	Adequacy of the follow‐up of the cohort.	Total quality score
Coppo et al. [15]	1	1	1	1	1	1	1	1	8
Dutt et al. [16]	1	1	1	1	2	1	1	1	9
Izquierdo et al. [17]	1	1	1	1	1	1	1	0	7
Jimenez et al. [18]	1	1	1	1	1	1	1	0	7
Volker et al. [19]	1	1	1	1	1	1	1	1	8
Gavriilaki et al.[25]	1	1	1	1	2	1	1	1	9
Prasannan et al. [22]	1	1	1	1	0	1	1	1	7

**TABLE 2:** Definition and criteria for the primary and secondary outcome following the administration of caplacizumab in TTP.

Outcome	Definition
Clinical Response	Sustained normalization of platelet counts >150 × 10^9^/L and of LDH <1.5 upper limits of normal after cessation of PEx
Exacerbation	**Clinical exacerbation of TTP is defined as the reappearance after a clinical response** and platelet count decreases to <150 × 10^9^/L and LDH is increased within 30 days of cessation of PEx/caplacizumab.
Refractoriness	Refractory TTP is persistent thrombocytopenia <50 × 10^9^/L and persistently raised LDH levels despite 5 PEx and steroid treatments. It is severe if thrombocytopenia remains <30 × 10^9^/L.
Relapse	Clinical relapse of TTP is defined as the reappearance after a clinical remission (defined as a sustained clinical response for at least 30 days after the last PEx/Caplacizumab) of a platelet count <150x109/L with other causes of thrombocytopenia ruled out, with or without clinical evidence of new ischemic organ injury.
5. Treatment Emergent Bleeding	Bleeding events after initiation of the treatment.
6. Major Bleeding	Major bleeding was defined as per the International Society of Thrombosis hemostasis bleeding scale guideline, which includes fatal bleeding and/or symptomatic bleeding in a critical area or organ and/or bleeding causing a decrease in Hb levels >2g.dl or leading of>2 units of blood transfusion.

### 3.3 Meta‐analysis

#### 3.3.1 Treatment‐emergent bleeding

**FIGURE 3:**
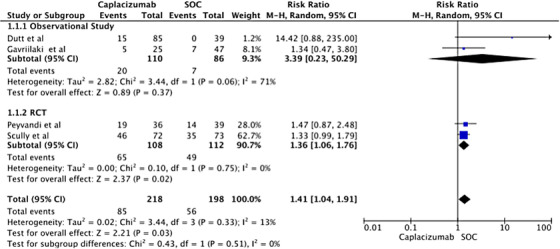
Forest plot showing risk ratio for bleeding in treating thrombotic thrombocytopenic purpura with Caplacizumab.

Four studies (two RCTs and two observational) that reported treatment‐emergent bleeding were analyzed for the risk associated with adding Caplacizumab compared to SOC at any time during follow‐up. The pooled RR shows a statistically significant increase in bleeding risk with Caplacizumab (RR 1.41, 95% CI: 1.04–1.91, *P* = 0.03). The overall analysis displayed a mild statistical heterogeneity (*I* ^2^ = 13%). Subgroup analysis indicated similar findings for the observational study group (RR 3.39; 95% CI: 0.23–50.29), while the RCT subgroup showed a statistically significant increased risk of bleeding with Caplacizumab (RR 1.36; 95% CI: 1.06–1.76) with no statical heterogenicity (*I* ^2^ = 0). Please refer to Figure 3 for the forest plot of the overall analysis.‘’

### 3.4 Major bleeding

**FIGURE 4:**
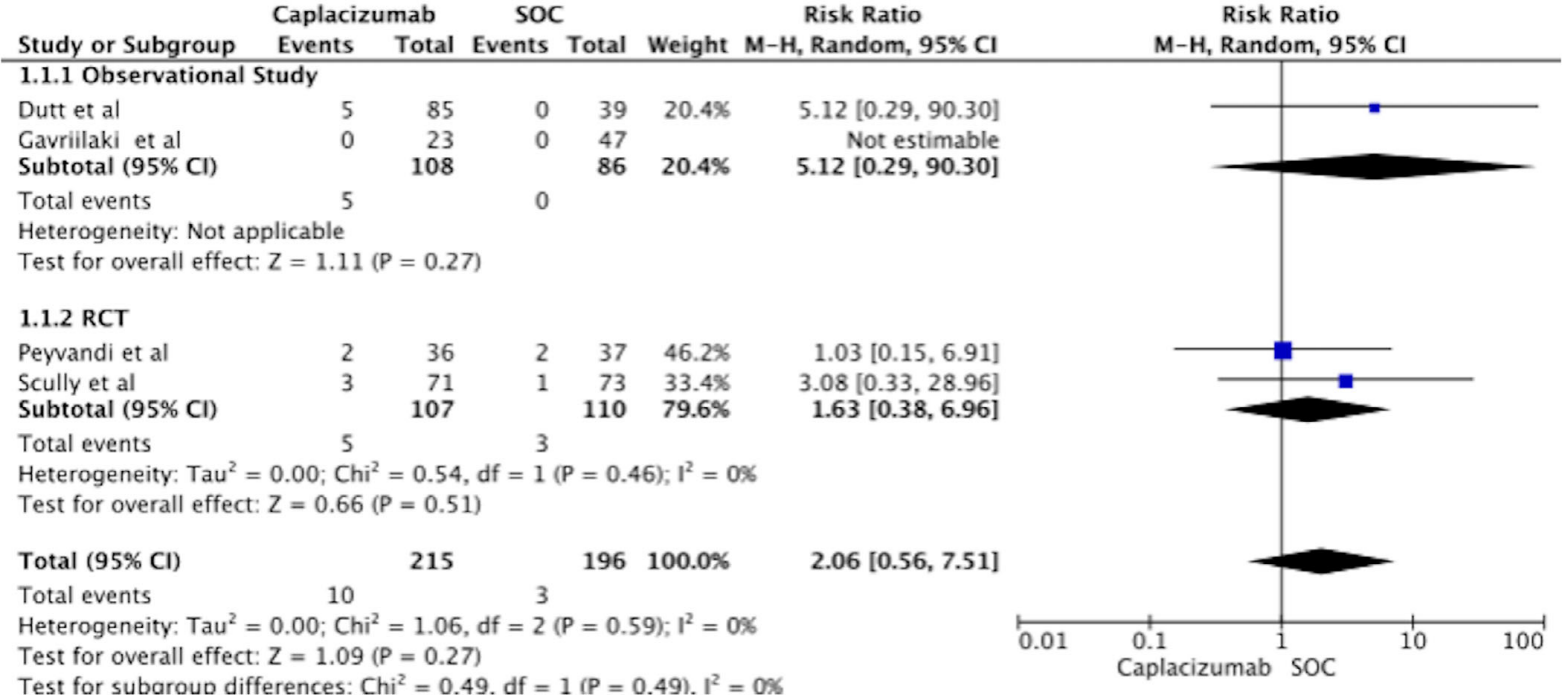
Forest plot showing risk ratio for major bleeding in treating thrombotic thrombocytopenic purpura with Caplacizumab.

Four studies (two RCTs and two observational) were included to analyze major bleeding. There was a non‐significant increase in major bleeding (RR: 2.08, 95% CI; 0.56–7.51) in the Caplacizumab group. Subgroup analysis by the study type also showed similar findings (Figure 4). Similarly, Table 3 Table 4summarizes the number, severity, and type of bleeding events in each study.

**TABLE 3:** Number of bleeding events in patients receiving caplacizumab, documented in the included studies.

Study	Bleeding Events	Major bleeding	Intracranial Bleeding
Scully et al. [4]	46	3	1
Peyvandi et al. [1]	19	2	1
Coppo et al. [15]	30	2	0
Volker et al. [19]	NA	NA	1
Jiminez et al. [18]	3	NA	
Dutt et al. [16]	15	5	2
Izquierdo et al. [17]	16	NA	2
Prasannan et al. [22]	NA	NA	NA
Gavriilaki [25]	12	0	0

**TABLE 4:** Descriptive characteristics of the included studies.

Study	Year/type/country of study	Sample size/median age I C		Gender female/male I C		No (%) Rituximab I C		No (%) ADAM TS13 < 10% vs. > 10% I C		No. (%) with neuro. Involvement on Presentation I C		Median follow‐up	First occurrence of TTP, *n* (%) I C	
Peyvandi et al. [1]	2016/RCT/International	36/41	39/42	24/12	20/19	NR	NR	28 (78)vs. 2[6]	30 (77) vs. 6[15]	NR	NR	1–12 month	24 (67)	27 (69)
Scully et al. [4]	2019/RCT/International	72/ 45	73/ 47	49/23	51/ 22	28 (39)	35 (48)	58 (81) vs. 13(180)	65 (89) vs 7[10]	6 [8]	5[7]	28‐days after treatment discontinuation	48 (67)	34 (47)
Coppo et al. [15]	2021/Obs/ France	90/ 45	180/43	63/27	127/53	90 (100)	123(68)	90 (100)	180 (100)	55 (61)	111(62)	127 days (range, 47–200 days)	34 (47)	NR
Dutt et al [16]	2022/Obs/UK	85/46	39/ 45	56/29	31/08	84 (99)	NR	84 (99) Vs.1[1]	NR	56 (66)	29 (74)	80 days (IQR, 59–166)	NR	NR
Jimenez et al [18]	2022/Obs/ Spain	10/43	20/40	9/1	14/06	**3 (30)**	**11 (55)**	10 (100)	20 (100)	3 (30)	8 (40)	37.5 days (IQR 32–39)	6 (60)	2 (90)
Izquierdo et al [17]	2022/Obs/ Spain	77/47	78/ 47	58/19	61/27	65 (84)	53 (68)	77 (100)	78 (100)	43 (56)	47 (60)	caplacizumab group was 216 days (IQR: 141–417 days) vs. 214 days (IQR: 138–467 days) in the non‐caplacizumab group	50 (65)	72 (92)
Gavriilaki et al [25]	2023/Obs/ Greece	23/47	47/47	15/8	33/14	16 (68)	15 (32)	23 (100)	47 (100)	11 (50)	25 (53)	19 months	NR	NR
Volker et al [19]	2023/Obs/ Germany	113/46	119/46	77/33	63/33	56 (80)	40 (95)	**110 (97)**	**93 (91)**	70 (100)	40 (93)	108.5 days (range, 5–330 days)	83 (73)	63 (53)
Prasannan et al. [22]	2023/Obs/ UK	64/41	50/45	22/42	13/37	61 (95)	43 (86)	NR	NR	NR	NR	35 days (range, 15–130)	59 (92)	46 (92)

Abbreviations: No, Number of patients; IQR, interquartile range; ObS, observational study; NR, not reported; NA, not available.

### 3.5 All‐cause mortality

**FIGURE 5:**
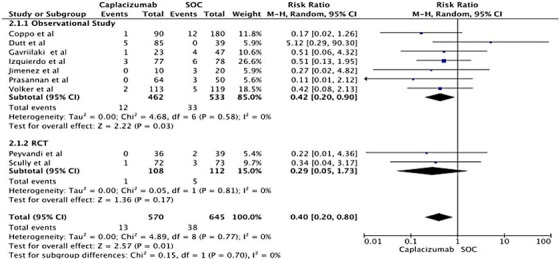
Forest plot showing risk ratio for mortality in treating thrombotic thrombocytopenic purpura with Caplacizumab.

Nine studies reported on all‐cause mortality, seven of which were observational studies, and two were RCTs. Comparing Caplacizumab with SOC, we found a statistically significant decrease in all‐cause mortality in the Caplacizumab group (RR 0.40, 95% CI: 0.20−0.80) with no statical heterogenicity (*I* ^2^ = 0). However, the subgroup analysis by study type revealed a nonsignificant decrease in mortality in RCT studies. Figure 5 demonstrates this forest plot of the overall analysis.

### 3.6 TTP relapse

**FIGURE 6:**
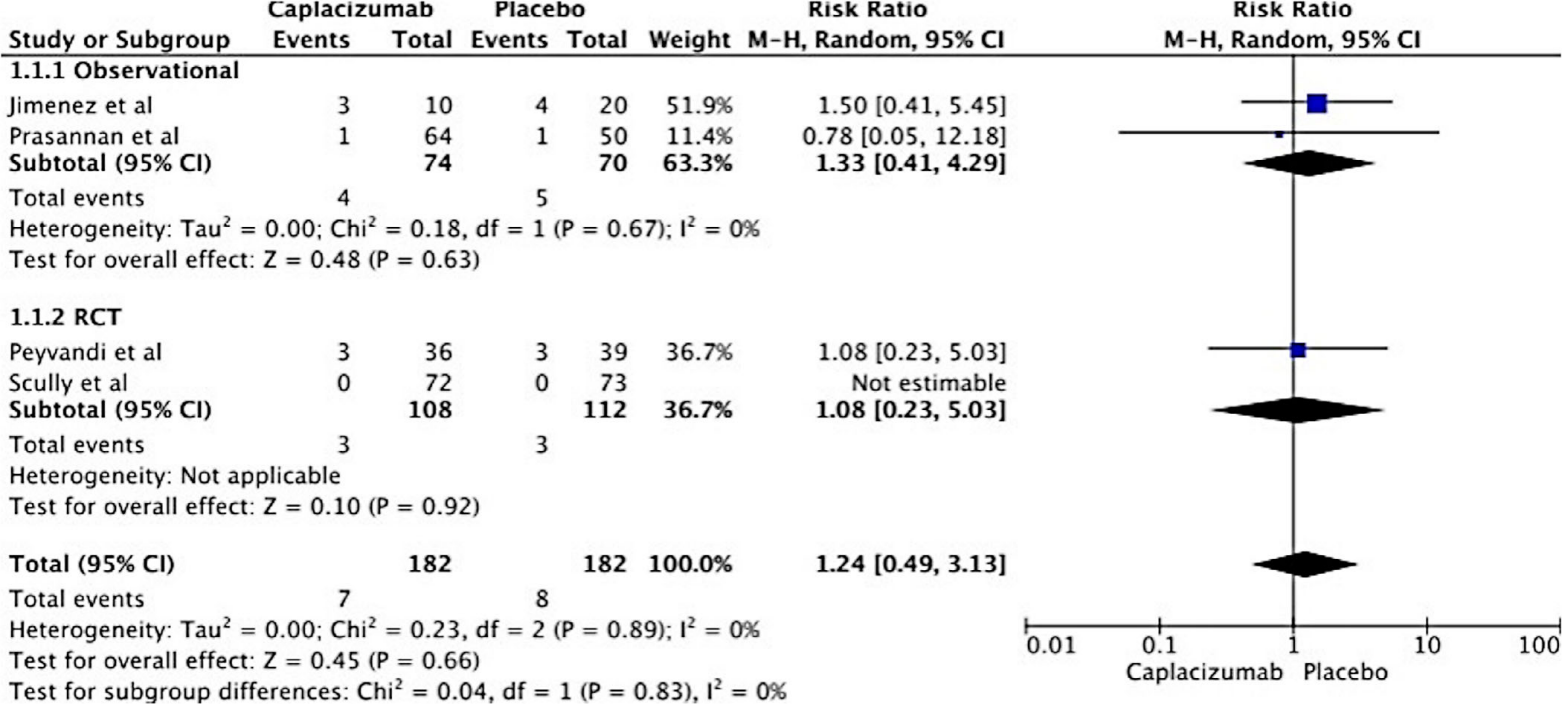
Forest plot showing risk ratio for relapse in treating thrombotic thrombocytopenic purpura with Caplacizumab.

Four studies reported TTP relapse rates: two observational and two RCTs. We observed a nonsignificant increase in the TTP relapse rate in the Caplacizumab group (RR 1.24, 95% CI: 0.49–3.13, *I* ^2^ = 0%, *P* = 0.66). This lack of heterogeneity was consistent across all studies (*I* ^2^ = 0%). Subgroup analysis yielded similar results: nonsignificant increases in TTP relapse rates for both observational studies and RCTs. Figure 6 displays the forest plot for the overall analysis. Figure S7 *displays risk ratio for relapse in treating thrombotic thrombocytopenic purpura with Caplacizumab based on old criteria*

### 3.7 Exacerbation

Five studies reported exacerbations (three observational and two RCTs). We found a significant reduction in the TTP exacerbation rate in the Caplacizumab group compared to the SOC group (RR 0.37, 95% CI: 0.15–0.94, *P* = 0.04), and the overall analysis had high statistical heterogeneity (*I* ^2^ = 76%). Subgroup analysis showed consistent results in the observational group but a nonsignificant decrease in exacerbation rate in the RCT group. Figure S1 displays the forest plot for the overall analysis. Figure S8 *displays risk ratio for exacerbation in treating thrombotic thrombocytopenic purpura with Caplacizumab based on old criteria*.

### 3.8 Refractory TTP

Six studies reported refractory TTP, four observational, and two RCTs. Comparing Caplacizumab with SOC, we found no significant difference in refractory TTP (RR 0.30, 95% CI: 0.07–1.18), and the analysis had high statistical heterogeneity (*I* ^2^ = 71%). Subgroup analysis revealed differing findings, with a nonsignificant decrease in the refractory rate of TTP in the observational study and a significant decrease in RCTs. Figure S2 demonstrates the forest plot of the overall analysis.

Figure S9 displays the forest plot showing risk ratio for refractory exacerbation in treating TTP with Caplacizumab based on old criteria.

### 3.9 Risk of thrombosis

**FIGURE 7:**
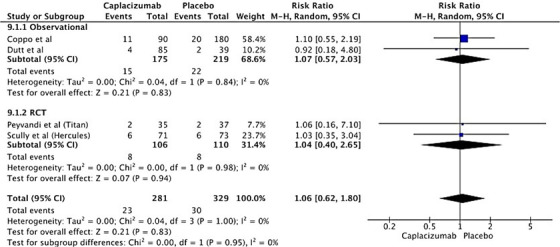
Forest plot showing risk ratio for risk of thrombosis in the treatment of thrombotic thrombocytopenic purpura with Caplacizumab.

Four studies are included (two observational and two RCTs) to find the risk of thrombosis. The risk of thrombosis was nonsignificantly increased across both subgroups (RR 1.06, 95% CI; 0.62–1.80, *P* = 0.83). Figure 7 shows the overall analysis of the study.

### 3.10 Time to normalization of platelet count

Eight studies assessed the time to platelet count normalization (six observational and two RCTs), Caplacizumab demonstrated a significant advantage over SOC. The overall analysis showed a significant reduction in time to response, with Caplacizumab leading by 2.26 days (WMD: −2.26, 95% CI: −3.26 to −1.26, *p* < 0.00001). High statistical heterogeneity was observed (*I* ^2^ = 95%). Subgroup analysis showed consistent results in both observational studies and RCTs. Figure S3 displays the forest plot for the overall analysis.

### 3.11 Duration of plasma exchange

Eight studies, comprising six observational and two RCTs, assessed the duration of plasma exchange. We observed a significant reduction in the mean duration of plasma exchange with Caplacizumab when compared to SOC. The overall analysis showed a significant decrease of 4.48 days (WMD: −4.48, CI: −5.68 to −3.27, *P* < 0.00001). Although there was high statistical heterogeneity (*I* ^2^ = 87%), subgroup analysis by study design consistently demonstrated shorter durations of PEx. Observational studies had a WMD of −4.82 days, and RCTs had a WMD of −3.67 days. See Figure S4 for the forest plot of the overall analysis.

### 3.12 Length of hospital stay

Six studies, one RCT, and five observational studies assessed the length of the hospital stay. Compared to SOC, Caplacizumab resulted in a shorter hospital stay of 3.65 days (WMD: −3.65, 95% CI: −7.35 to −0.05, *P* = 0.05). While there was substantial statistical heterogeneity (*I* ^2^ = 88%) in the overall analysis, subgroup analysis by the study design consistently showed shorter hospital stays [observational studies: WMD: −3.69; RCTs: WMD: −3.53]. Refer to Figure S5 for the forest plot of the overall analysis.

### 3.13 ICU stay

Four studies (1 RCT and 3 observational) reported the mean difference in the length of ICU stay. Compared to SOC, Caplacizumab resulted in a significantly shorter ICU stay by 2.24 days (WMD: −2.24, CI: −4.16 to −0.33, *P* = 0.05) with high statistical heterogenicity (*I* ^2^ = 94%). Subgroup analysis according to the study design revealed differing findings. In the RCT, the Caplacizumab group showed a significant reduction in ICU stay by 10.25 days, while in observational studies, the reduction was nonsignificant (WMD: −0.62). See Figure S6 for the forest plot of the overall analysis

## 4 DISCUSSION

Despite proven benefits in the management of TTP, there has been some controversy among experts over the net benefit of Caplacizumab use alongside SOC in the disease. This has been mainly attributed to the cost of the drug, questionable effects on mortality, and increased relapse and bleeding risk [20]. In this comprehensive systematic review and meta‐analysis comparing the risk of clinical recurrence and major bleeding of adding Caplacizumab to SOC with SOC alone in the management of immune TTP, we found a statistically significant decrease in the risk of TTP exacerbations and a nonsignificant increase in the risk of relapse, while there was a nonsignificant increase in major bleeding with Caplacizumab use. Due to the rarity of TTP and the paucity of clinical trials, we elected to utilize observational studies alongside RCTs, totaling seven observational studies and two RCTs.

The elevated risk of relapse in TTP patients receiving Caplacizumab was initially documented in the phase II TITAN trial [1]. During the designated follow‐up period, eight patients experienced relapse within the first month after discontinuing Caplacizumab, while no patients in the SOC group encountered a relapse. Importantly, a majority of the relapsed patients exhibited an ADAMTS13 activity level below 10% at the time of discontinuing Caplacizumab, underscoring the fact that Caplacizumab does not specifically target the underlying pathophysiology responsible for triggering TTP. Subsequent efforts were made to address this concern in the phase III HERCULES trial [21], where patients were permitted to continue treatment until the resolution of the ADAMTS13 activity deficit. Although the implementation of this strategy resulted in lower reported relapse rates, it was still evident that relapse occurrences remained higher in the Caplacizumab group compared to those receiving SOC alone.

Caplacizumab, given its mode of action of abolishing microthrombi formation, has a temporizing effect on platelet count during the period of the treatment. Consequently, a limitation arises when evaluating exacerbations and relapses using the conventional 30‐day post‐ PEx cut‐off for a durable remission, as Caplacizumab is typically administered for a duration of 30 days. New criteria were proposed by the International TTP Working Group in 2021 to avoid misrepresentations of clinical recurrences with Caplacizumab in addition to the SOC. We were able to utilize data on the timing of clinical recurrences from the two trials and three observational studies to re‐allocate patients into the relapse/exacerbation outcome using the new definition, as most studies utilized the old definition of outcomes for reporting.

A recent meta‐analysis by Djulbegovic et al. [10] addressed the potential benefits of Caplacizumab over TTP; however, the old criteria of exacerbation and relapse were implemented, as used in the original studies, which has resulted in differences between their and our findings. Contrary to the findings of Djulbegovic et al. [10], reporting a significant increase in the relapse rate with Caplacizumab (RR 3.81; 95% CI, 1.58–14.28), we found a nonsignificant increase in relapse rates in both study design groups (RR 1.24, 95% CI: 0.49–3.13). The beneficial effect of Caplacizumab on exacerbation rate, albeit significant, was less profound in our study (RR 0.37 (0.15–0.94) compared to 0.16 (0.07–0.47) in the study by Djulbegovic et al.). We were able to re‐allocate the population as per the revised IWG criteria, where the cessation of PEx‐Rituximab/Caplacizumab has been the baseline for the determination of exacerbation and relapse. The findings from our study, therefore, consider the temporizing effect of Caplacizumab on platelet count alongside that of PEx, generating revised pooled evidence on the safety of the supplementation of the anti‐von Willebrand factor (VWF) therapy in TTP.

In theory, the use of Caplacizumab allows a swifter clinical response and allows more time for other therapeutic modalities like rituximab and corticosteroids to target the underlying immune phenomenon. However, a recent report by Prasannan et al. [22] found delayed ADAMT13 recovery in a cohort treated with Caplacizumab and SOC compared to SOC alone, suggesting the possibility of an unidentified process affecting durable response, or perhaps, the earlier stoppage of TPE might be contributing. Given the relatively lower recurrence rates in TTP since the incorporation of Rituximab in SOC, further studies with larger sample sizes are required to elucidate the effect of Caplacizumab on TTP recurrence. Supplementation of antithrombotic drugs, including low‐dose aspirin alongside PEx‐Caplacizumab to enhance the therapeutic efficacy on long‐term outcomes in TTP has been proposed by a few practice guidelines; however, limited studies have been performed on the additional risk of bleeding in this scenario given that patients treated with Caplacizumab already possess higher risk [23].

Our study showed an increased incidence of bleeding in the RCT subgroup only but not the overall analysis. We additionally investigated the risk of major bleeding, as the degree of bleeding is important to consider. We found a nonsignificant increased risk of major bleeding with the Caplacizumab group at a RR of 2.08. Most bleeding events reported were mild to moderate and did not require therapeutic interventions. It should also be noted that there is systematic under‐reporting of bleeding in historical TTP cohorts, as thrombotic events are likely more prevalent. Bleeding events are summarized in Table 1. Decreased vWF levels seen with Caplacizumab, coupled with thrombocytopenia from TTP, predispose to the increased bleeding risk in these patients. This increase in minor bleeding may be considered a reasonable trade‐off for a quicker recovery and reduced chance of TTP exacerbation, as these latter outcomes could potentially be more problematic than the mild bleeding side effects. Nevertheless, case reports of severe bleeding complications like cerebral hemorrhage exist in the literature and warrant judicious use of the medication in patients with a high risk of bleeding [8, 24].

Our overall analysis found a statistically significant reduction in mortality in the Caplacizumab group compared to the SOC group, but these results were not replicated in the subgroup analysis by study design. A similar nonsignificant reduction in mortality was found in a recent meta‐analysis by Djulbegovic et al. that included three observational studies and two major trials [10]. An integrated analysis by Peyvandi et al. of the two trials did show a statistically significant reduction in TTP‐related mortality [1]. It is to be noted that the number of mortality events in both arms of the RCTs was lower than the reported average in the literature. Our study also showed a significant reduction in hospital stay by 5.14 days with Caplacizumab compared to the SOC. This was consistent in the subgroup analysis as well. The Djulbegovic et al. study showed a similar significant reduction in the hospital stay by 6 days with Caplacizumab. There was no significant difference in the mean days of ICU stay between the Caplacizumab and placebo groups; however, there was a significant reduction in ICU stays in the RCT subgroup by 2 days [10]. Organ involvement is important to consider in assessing the mortality risk, and it should be noted that only the HERCULES trial stratified patients with and without neurologic involvement during randomization, using baseline Glasgow Coma Scale score of ≤12 versus > 13.

Contrary to the findings of Djulbegovic et al., our analysis did not reveal a significant decrease in TTP refractoriness. It is unclear if Caplacizumab alters the long‐term disease course in TTP, given that it does not correct the underlying pathology of the auto‐antibody production. We found that Caplacizumab significantly decreased the time‐to‐response by 2.85 days in the overall analysis, and this result was comparable to previous studies. The shortened time‐to‐platelet‐normalization may have reduced TTP duration by 3.63 days, as we discovered in our meta‐analysis. The use of Caplacizumab in RCTs compared to SOC showed inconclusive results for the risk of thrombosis, with an RR of 1.02 and an absolute risk of 0%. Our study demonstrated no effect on the risk of thrombosis like the Djulbegovic et al. study that also showed no difference in risk of thrombosis between the two groups [10].

Our study has several strengths, including the number of studies covered. We divided our analysis into subgroups according to study design types and calculated the analysis’ combined estimate. We also extensively focused on the side effects, like bleeding, relapse, and exacerbation, based on the latest guidelines. The IWG 2021 revised criteria are yet to be validated prospectively. Our findings, therefore, not only add directions off the controversiality of Caplacizumab on TTP but also supply evidence to the IWG regarding the pooled implications of their revised criteria.

Our study has several limitations. First, despite our thorough and methodical literature search, only a few papers matched the inclusion criteria. Second, several of the same patients were enrolled in multiple of the included trials. We did not investigate whether Caplacizumab is cost‐effective, which is vital for deciding which treatment to utilize. Most of the observational studies did not report bleeding in the control group. We conducted the sub‐group analysis based on the study design but could not stratify the analysis based on the ADAMTS13 activity. There was also variability in rituximab and corticosteroid use between groups and variability in the timing of Caplacizumab treatment as well as the duration of TPE between the earlier studies and the HERCULEs study. We were also unable to assess the impact difference when caplacizumab is used as initial treatment versus in recurrence/refractoriness as the clinical trials did not report this subgroup analysis of outcomes. The retrospective study by Izquierdo et al. compares the subgroup of patients treated with caplacizumab as initial treatment (*n* = 44) versus patients treated with caplacizumab only after an exacerbation or refractoriness. The median time to clinical response was shorter when caplacizumab was used initially, and there were also lower incidences of exacerbation and refractoriness. Finally, the studies’ follow‐up periods varied and were brief in some of the observational studies.

## 5 CONCLUSION

In this systematic review and meta‐analysis, we found a non‐statistically significant increase in the relapse rate and major bleeding with Caplacizumab use compared to SOC alone, as well as a statistically significant modest decrease in the risk of exacerbations. The ideal way to utilize Caplacizumab remains a topic of debate; however, our results suggest that Caplacizumab is relatively safe for the treatment for aTTP as regards recurrence and bleeding risk, albeit with modest improvement in major patient outcomes. The benefits of Caplacizumab in the acute stage of the disease, in our opinion, warrant consideration of its use, especially in severe organ involvement in TTP. To direct this further, larger studies with extended follow‐up periods are required to further delineate the overall cost‐benefit and bleeding risk of Caplacizumab in TTP compared to the SOC. Future research should also control for various confounders like steroids and rituximab, as well as re‐assess the ideal TPE stoppage strategy in Caplacizumab patients, which might be impacting the overall outcomes in this new era.

## AUTHOR CONTRIBUTIONS

**Niraj Neupane**: Conceptualization; data extraction; manuscript writing; editing. **Sangharsha Thapa**: Conceptualization; data analysis; manuscript writing. **Amir Mahmoud**: Manuscript writing. **Abhinav Bhattarai**: Manuscript writing and editing. **Anil KC**: reviewing and editing. **Shreeja Shikhrakar**: reviewing and editing. **Sayuri Gurusinghe**: reviewing and editing. **Peter Kuiodes**: Supervision; reviewing; editing; and approval.

## CONFLICT OF INTEREST STATEMENT

The authors declare no conflict of interests.

## FUNDING INFORMATION

The study received no funding.

## ETHICS STATEMENT

The authors have confirmed that ethical approval statement is not needed for this submission.

## PATIENT CONSENT STATEMENT

The authors have confirmed that patient consent statement is not needed for this submission.

## Supporting information

Supporting Information

## Data Availability

All the required data are in the manuscript itself.
