# Supplementary material for: Does Caplacizumab for the management of thrombotic thrombocytopenic purpura increase the risk of relapse, exacerbation, and bleeding? An updated systematic review and meta‐analysis based on revised criteria by the International Working Group for thrombotic thrombocytopenic purpura
Source: EJHaem. 2023 Dec 18;5(1):178–90. doi: 10.1002/jha2.833 (PMC10887272; doi:10.1002/jha2.833)
Supplement: Supplementary file 1 — Supporting Information [file JHA2-5-178-s001.docx]

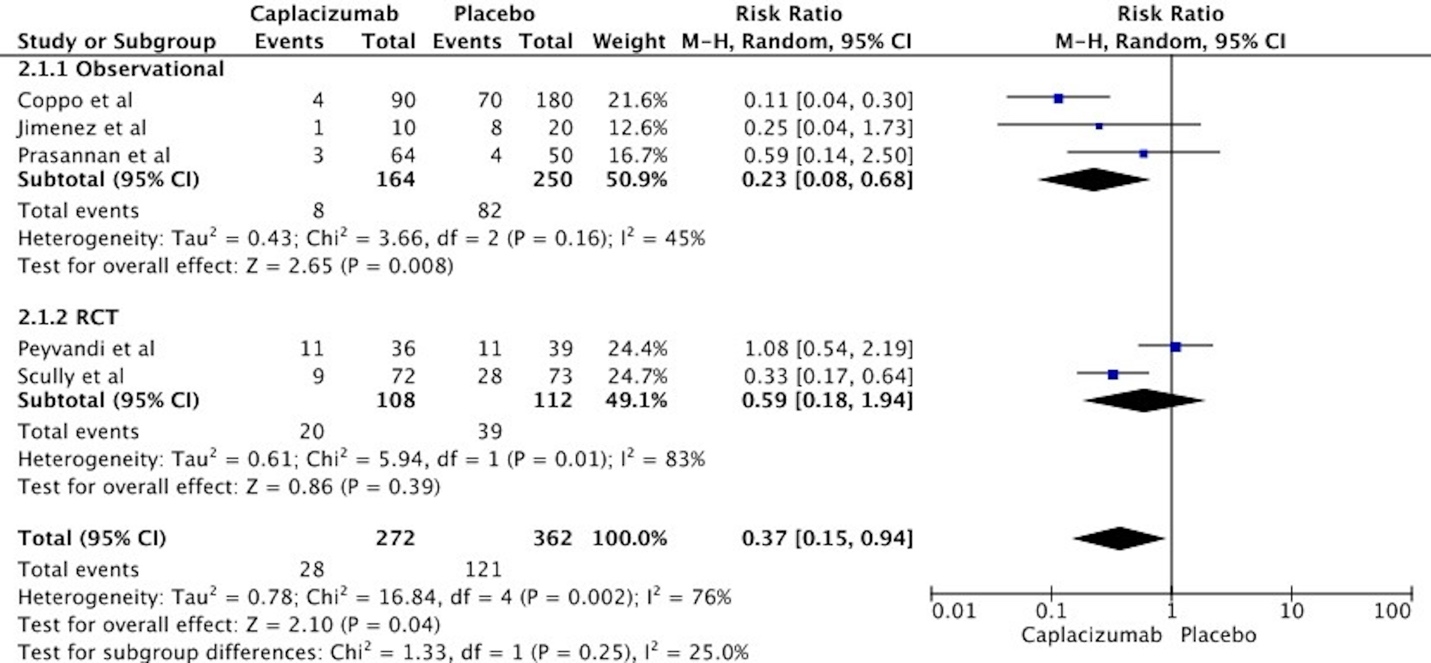


***Figure S1 : Forest plot showing risk ratio for exacerbation in treating thrombotic thrombocytopenic purpura with Caplacizumab.***


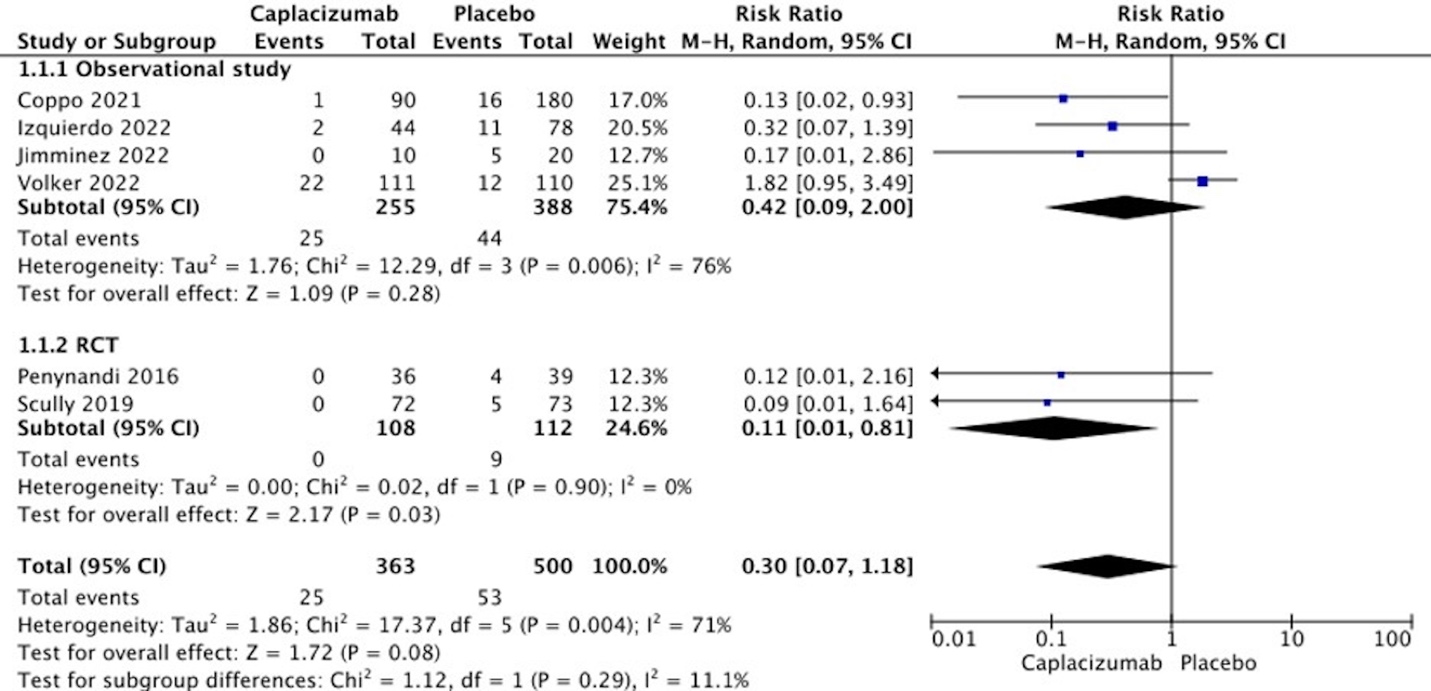


***Figure S2: Forest plot showing risk ratio for refractory exacerbation in treating thrombotic thrombocytopenic purpura with Caplacizumab.***

.


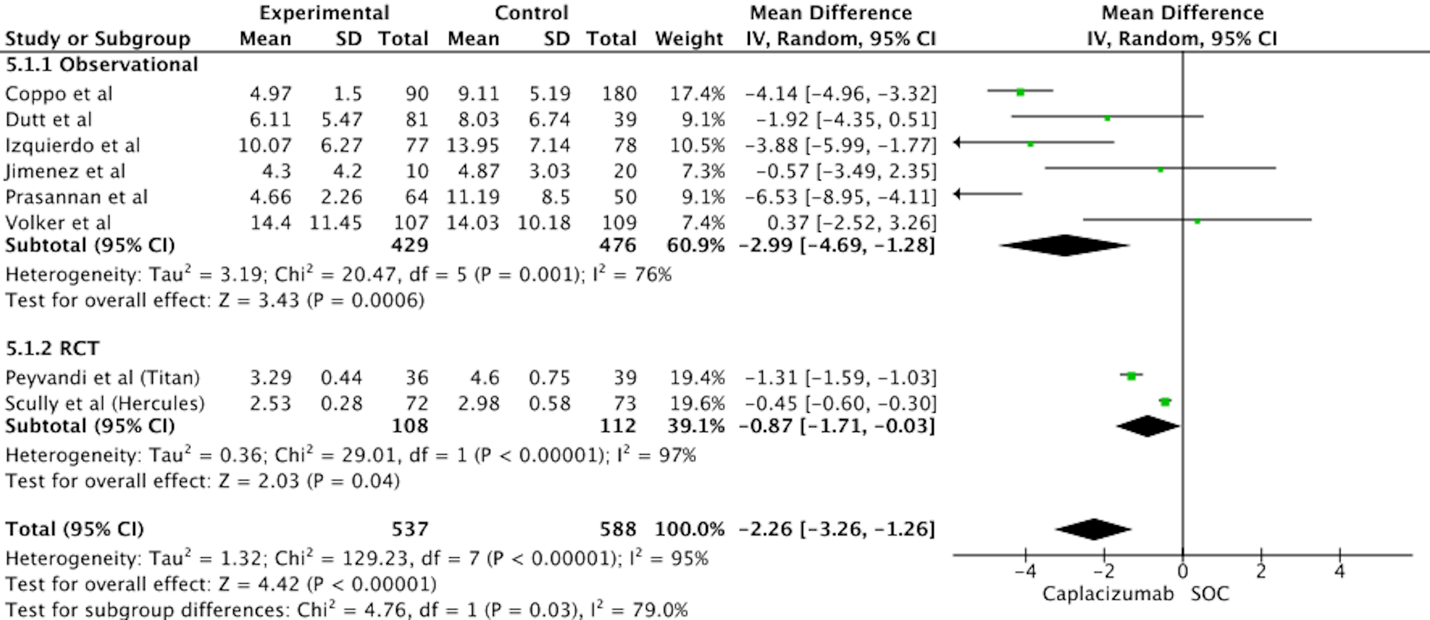


***Figure S3: Forest plot showing WMD of platelet counts normalization time in treating thrombotic thrombocytopenic purpura with Caplacizumab.***


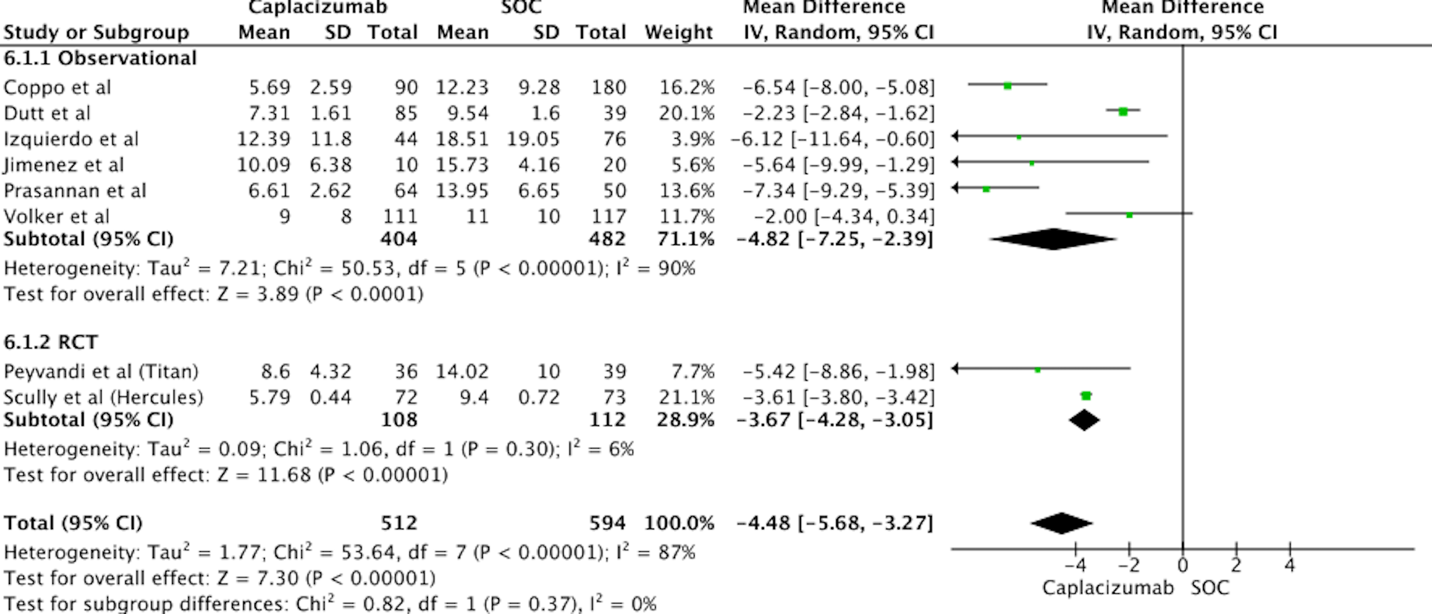


***Figure S4: Forest plot showing WMD of plasma exchange time in treating thrombotic thrombocytopenic purpura with Caplacizumab.***

***Figure S5: Forest plot showing WMD of hospital stay treating thrombotic thrombocytopenic purpura with Caplacizumab.
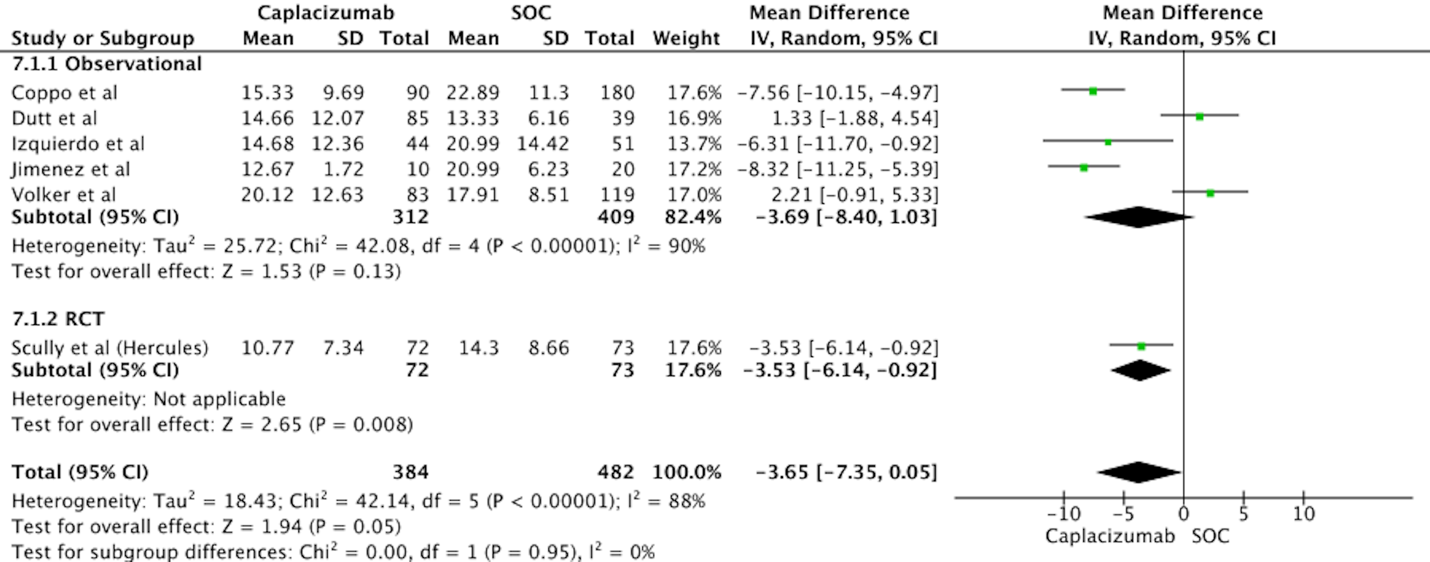
***

.

***
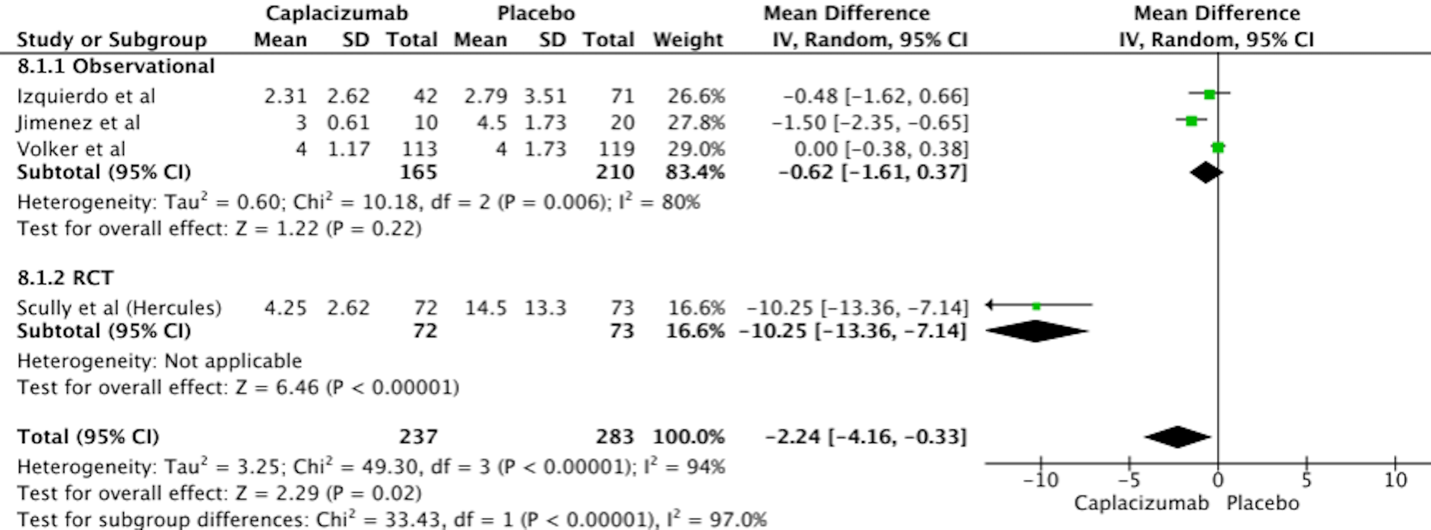
 Figure S6 : Forest plot showing WMD of ICU stay treating thrombotic thrombocytopenic purpura with Caplacizumab. WMD, weighted mean difference.***


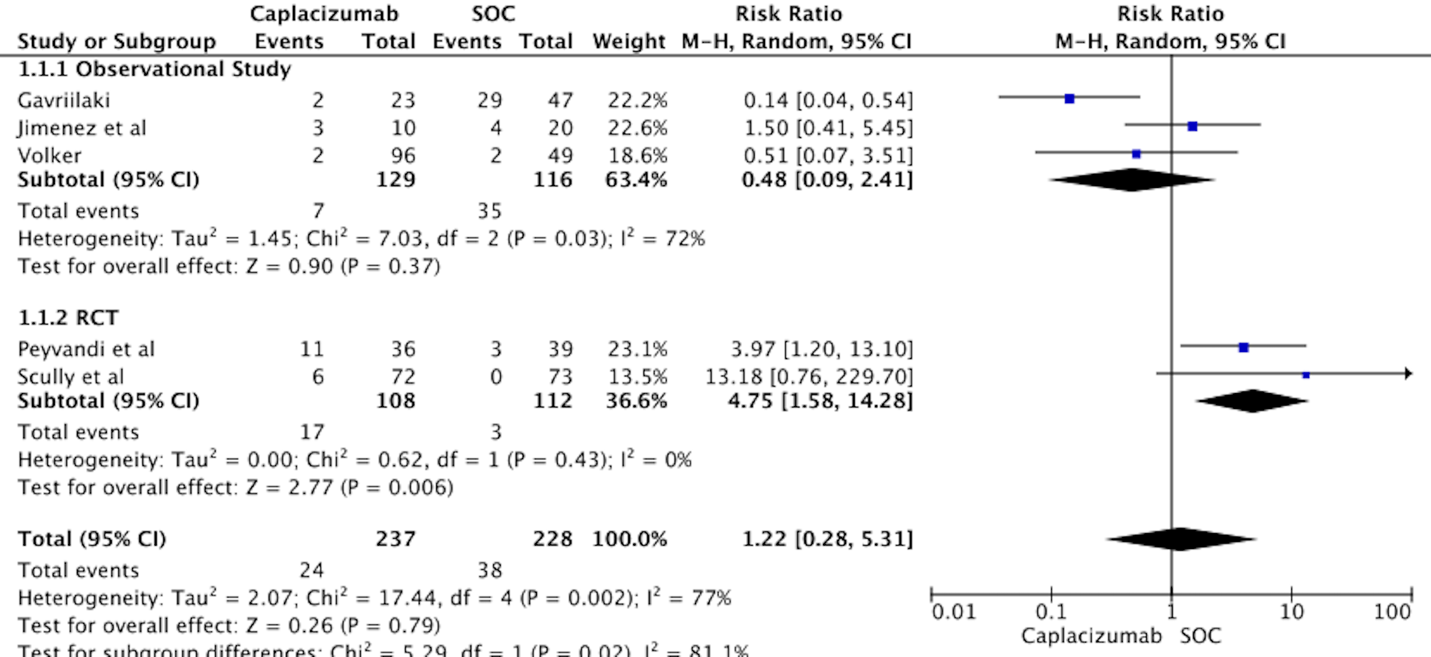


***Figure S7: Forest plot showing risk ratio for relapse in treating thrombotic thrombocytopenic purpura with Caplacizumab based on old criteria.***

***
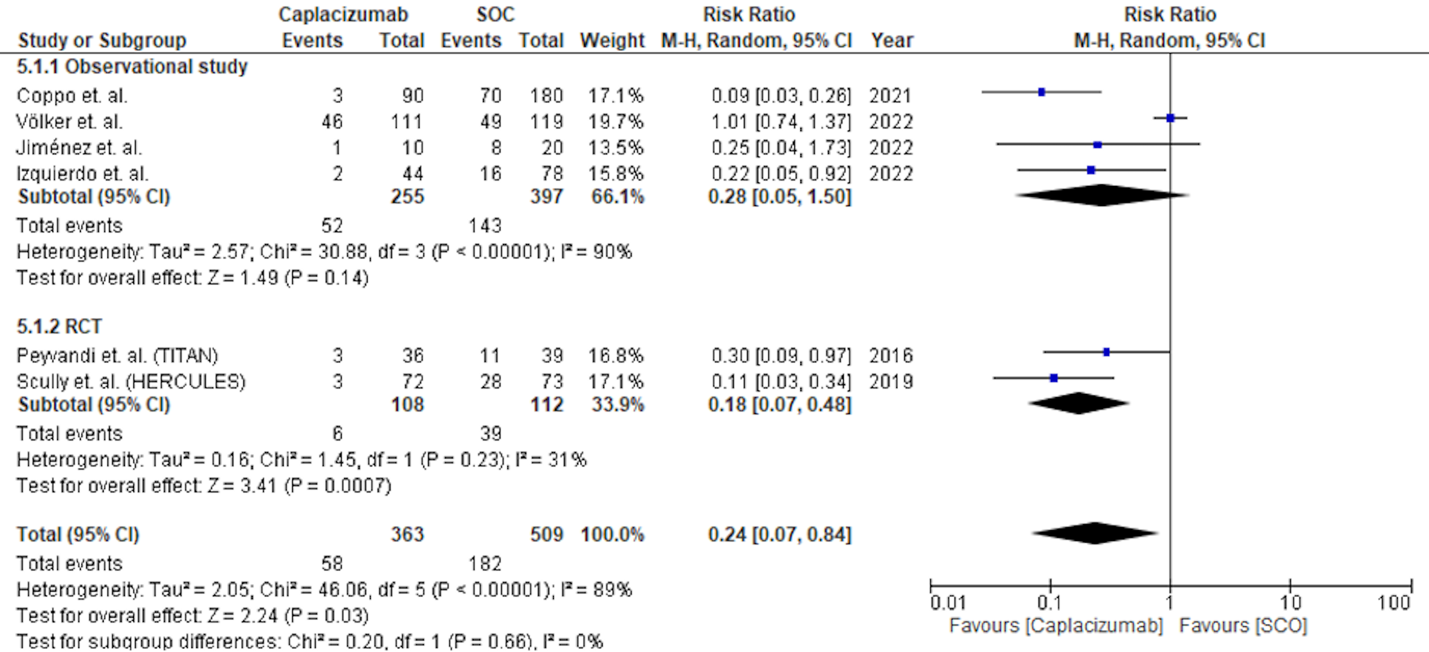
 Figure S8: Forest plot showing risk ratio for exacerbation in treating thrombotic thrombocytopenic purpura with Caplacizumab based on old criteria***


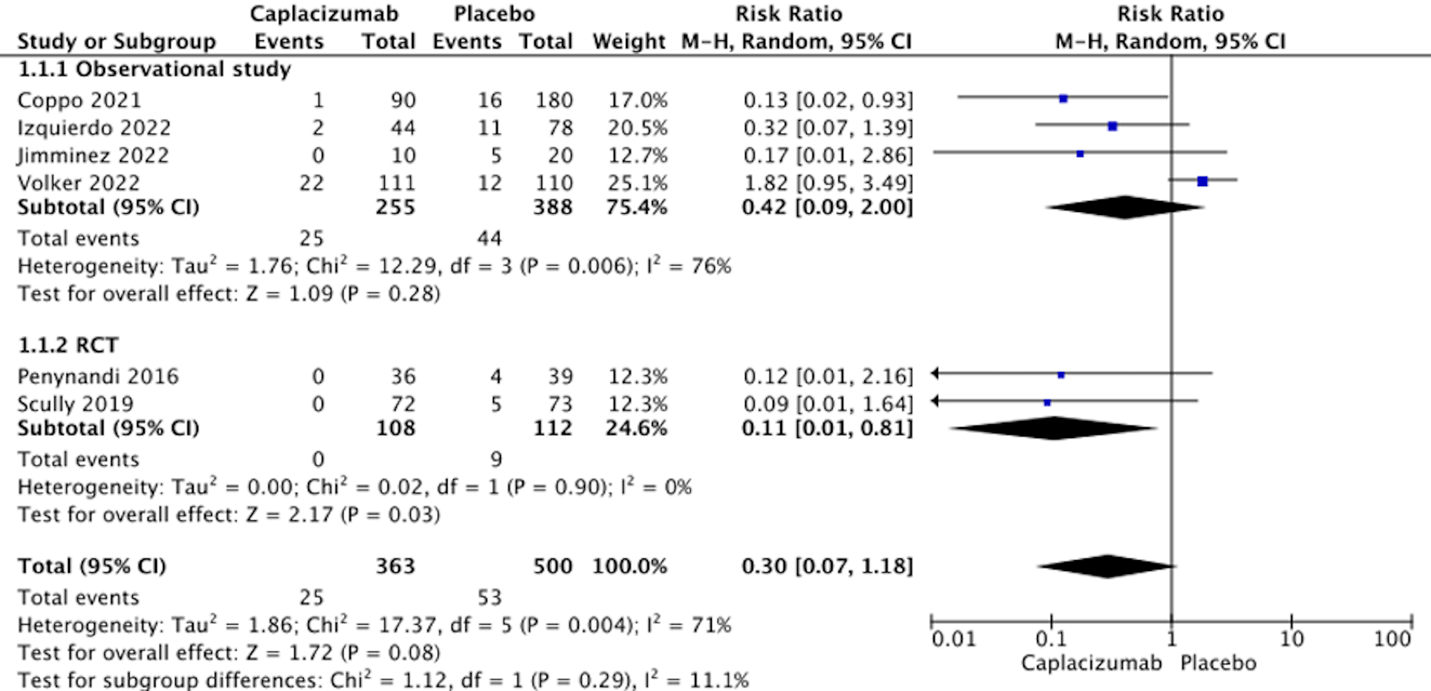


Figure S9: ***Forest plot showing risk ratio for refractory exacerbation in treating thrombotic thrombocytopenic purpura with Caplacizumab based on old criteria***
